# Evaluation of *Urtica dioica* and *Phillyrea latifolia* extracts as feed additives for enhancing innate immunity in gilthead seabream (*Sparus aurata* L.)

**DOI:** 10.1007/s10695-026-01714-z

**Published:** 2026-05-27

**Authors:** Osman Nezih Kenanoğlu, Soner Bilen, Sevdan Yılmaz

**Affiliations:** 1 Department of Food Engineering, Faculty of Engineering and Architecture, Kastamonu University, Kastamonu, Turkey; 2 Department of Veterinary, İhsangazi Vocational School, Kastamonu University, Kastamonu, Turkey; 3 Department of Aquaculture, Faculty of Marine Sciences and Technology, Çanakkale Onsekiz Mart University, Çanakkale, Turkey

**Keywords:** *Sparus aurata*, Immunostimulation, *Phillyrea latifolia*, *Urtica dioica*, Cytokine expression

## Abstract

This study was aimed at determining the effects of ethanolic extracts of nettle (*Urtica dioica*) and green olive tree (*Phillyrea latifolia*), used as feed additives, on innate immune responses in gilthead seabream (*Sparus aurata*). Ethanolic extracts from leaves of both plants were incorporated into feeds in two separate experimental setups, and the fish received 0, 50, 100, 200, and 400 mg/kg nettle or green olive tree extracts for 60 days. Growth performance was evaluated on the 30th and 60th days, and hematological parameters as well as the immune responses were assessed on the 60th day. Moreover, the nettle group was challenged with *Vibrio anguillarum* at the end of the 60th day. Supplementation at different doses increased lysozyme and myeloperoxidase activities significantly in both trials (*p* < 0.05). Respiratory burst activity also significantly increased after nettle supplementation (*p* < 0.05). It was determined that nettle supplementation resulted in 96% survival rate after the challenge test. In both trials, expression levels of *IL-1β*, *IL-6*, *IL-10*, *IL-18*, and *TNF-α* genes were analyzed and evaluated alongside other parameters. Consequently, the findings indicated that nettle, particularly at 100 mg/kg feed, provided the strongest support for immunostimulation and disease resistance in gilthead seabream. In contrast, green olive tree extract showed preliminary immunomodulatory effects that require further confirmation by deeper phytochemical standardization/quantitative characterization and a valid pathogen challenge.

##  Introduction

Fish are a rich source of protein, essential amino acids, and unsaturated fatty acids. These features make them a significant part of human nutrition. Due to intensive fishing and the depletion of wild stocks, approximately half of the fish consumed today are produced through aquaculture. Aquaculture has been the fastest-growing food sector for many years. One of the various fish species cultured is the gilthead seabream (*Sparus aurata*). According to the latest available data, global gilthead seabream aquaculture production in 2022 was 346,000 tonnes (FAO 2025). Likewise, it constitutes a significant portion of Turkish aquaculture. In 2022, Turkey produced 152,469 tonnes of gilthead seabream through aquaculture, making it the world's leading producer (TÜİK 2025).

Although, particularly during its early life stages, *S. aurata* enters brackish environments, it is a carnivorous euryhaline fish that predominantly inhabits saltwater. Aquaculture of *S. aurata* is favored for several reasons, including high market value and demand, rapid growth rate, euryhaline characteristics, adaptability, satisfactory survival rate, established aquaculture technology and expertise, efficient feed conversion rate, and tolerance to a broad range of temperatures. However, as with many fish species, problems appear in gilthead seabream farming. One of which is the use of drugs to combat diseases. Antibiotics, in particular, pose significant environmental and health concerns, such as the emergence of bacterial resistance and residues in fish meat. Despite having partial solutions to these problems, researchers are still seeking better alternatives. In this context, a growing number of studies have investigated the potential use of whole plants, plant parts, or their derivatives (extracts, oils, specifically isolated compounds, etc.) as feed additives to boost fish immunity. Most of these studies suggest that such products may represent viable alternatives. For example, it has been shown that a mixture of sage (*Salvia officinalis*) and spirulina (*Arthrospira platensis*) positively influences lysozyme, nitric oxide, and immunoglobulin M (IgM) levels in Nile tilapia (Abdellatief et al. 2018). Likewise, rosemary (*Salvia rosmarinus*) leaf was demonstrated to favorably affect Nile tilapia’s catalase, lysozyme and complement activities (Naiel et al. 2020). In another investigation, Dawood et al. (2020) reported that menthol essential oil supplementation caused a significant decrease in the gene expression of proinflammatory cytokines (*IL-1β* and *IL-8*) in the gills, liver and intestines of some teleost fishes and a significant increase in catalase and superoxide dismutase activities. Salomón et al. (2020), who emphasized that essential oils have a prophylactic effect, stated that sage (*S. officinalis*) and lemongrass (*Lippia citriodora*) leaf extracts activated the expression of genes related to humoral immunity and inflammation, leukocyte cell receptors and antioxidant enzymes in seabream. It has been reported in the literature that the citral component found in citrus and lemongrass oils has anti-inflammatory and antibacterial effects due to its monoterpenoid structure (Ponce-Monter et al. 2010; Silva-Angulo et al. 2015) and also plays a beneficial role in stimulating some immune-related indicators, including lipoperoxidation and antioxidant enzyme levels (Mori et al. 2019). As inferred from the literature summary above, plant extracts or other plant-derived products could be viable alternatives to chemical drugs. Herbal bioactive compounds present in these natural products may exhibit antibacterial, antiviral, and antifungal functions and could increase resistance against infectious microorganisms (Burdock and Carabin 2004; Citarasu 2010). Moreover, these products are typically considered cost-effective and environmentally safe (Reverter et al. 2014).

Nettle (*Urtica dioica*) is a perennial plant species belonging to the Urtica genus of the Urticaceae family (Ahmed and Parsuraman 2014). It is found in temperate regions of Europe, Asia, North Africa, and North America at altitudes up to 1800 m (Grauso et al. 2020). Nettle, which is found naturally on the edges of fields, roads, and forests, is very rich in terms of chemical content. The chemical properties of the above and below ground parts of the nettle are different from each other, and it contains nearly 20 chemical substances in its structure (lectins, amines, fatty acids, sterols, polyholozides, etc.) (Ayan et al. 2006). Due to its rich chemical content, nettle has been used widely in the medicine, food, fiber, paint and cosmetic industries for centuries. Green olive tree (*Phillyrea latifolia*), which is one of the three species (*P. media*, *P. angustifolia*, and *P. latifolia*) in the Phillyrea genus, belongs to the Oleaceae family. It is an evergreen small tree that is common in the Mediterranean climate zone (Yazici-Tutunis et al. 2016). *P. latifolia* also contains a broad range of phytochemicals, including phenolic compounds, flavonoids, iridoids, triterpenoids, lignans, and phenylpropanoids (Uysal 2020). It has been suggested that *P. latifolia* can be utilized as a natural antioxidant (Uysal 2020). Despite having such rich phytochemical contents and common utilization in various fields, the effects of *U. dioica* and *P. latifolia* in gilthead seabream remain unexplored. Therefore, we hypothesized that utilization of these plants as feed additives would be beneficial for enhancing immunity. In light of this information, this study aimed to investigate the applicability of nettle and green olive tree as immunostimulants in seabream. To test this, we supplemented the fish with the ethanolic extracts of these plants and examined the changes in growth performance, hematological parameters and immune status.

## Materials and methods

The study protocol was approved in advance by the Çanakkale Onsekiz Mart University Local Ethics Committee of Animal Trials (Decision no: 2021/02–04).

### Materials

Experimental animals (Gilthead seabream, *Sparus aurata*) were acquired from “Çanakkale İda Food and Aquaculture Production Co. Ltd.” Three hundred and forty-five fish were supplied for each experiment (A total of 690 fish) at three-month intervals. For both trials, the fish were provided from the same facility. The nettle and green olive tree plants were acquired from a local company (Botanikmarket; Köprübaşi-Ordu, Turkey). Fish were fed with a commercial trout feed (Alltech Coppens®; Table 1).

**Table 1:** Composition of the commercial feed used in the study

Basic nutritional values	Amount
Crude protein	56.0%
Crude oil	15.0%
Crude cellulose	0.2%
Ash	13.0%
Calcium	3.1%
Phosphorus	1.89%
Sodium	0.90%
Vitamins and microelements	
Vitamin A	14,000 IU/kg
Vitamin D_3_	1542 IU/kg
Manganese	26 mg/kg
Zinc	78 mg/kg
Iron	78 mg/kg
Iodine	65 mg/kg
Copper	65 mg/kg

### Methods

**Fig. 1:**
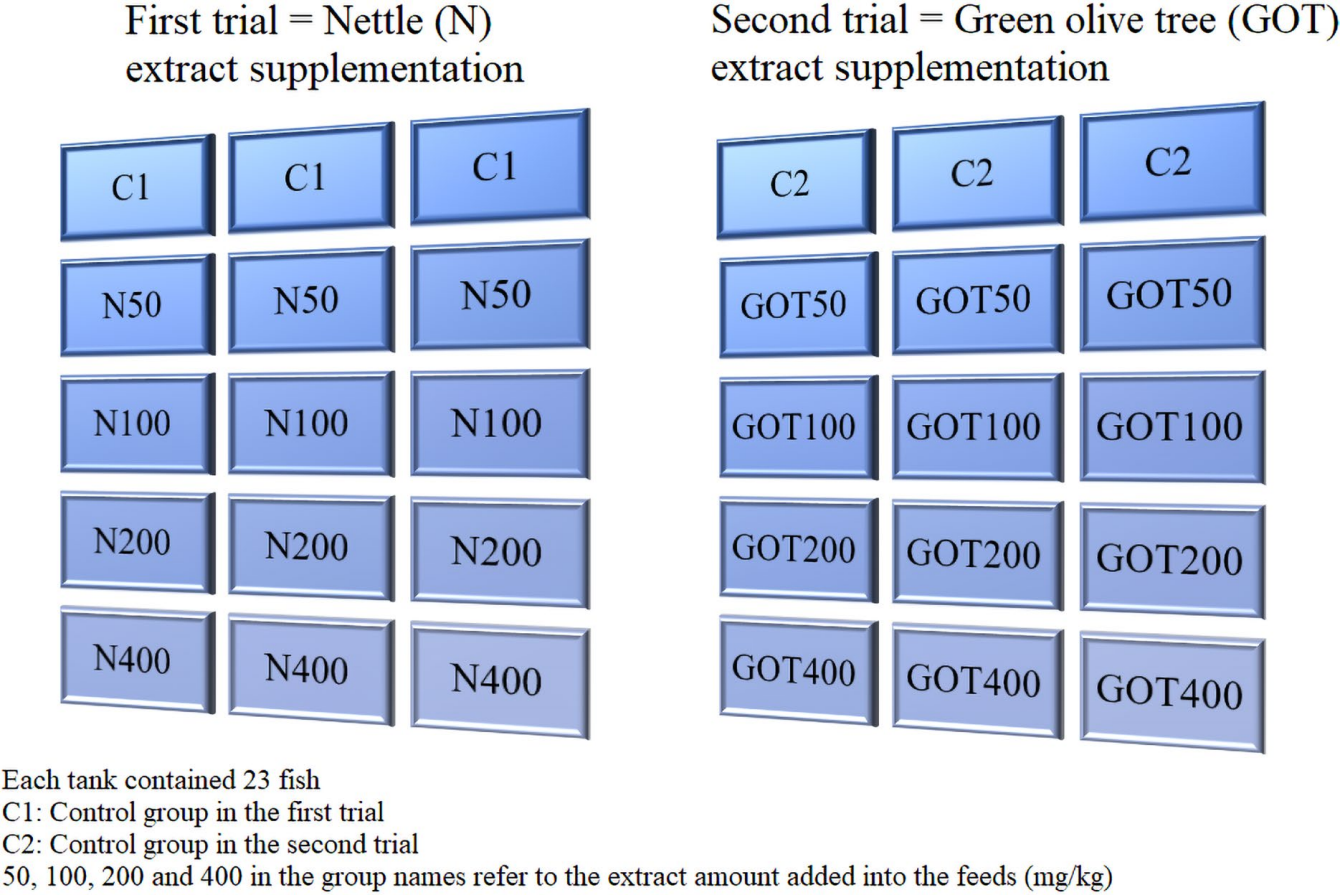
Schematic demonstration of the experimental design

Fish were acclimated to the laboratory conditions for 15 days upon arrival. Each tank contained 23 fish, accounting for a total of 69 fish per treatment with the triplicate experimental design (Fig. 1). Both trials were carried out by following the same procedures, except that the extracts administered were different, and a challenge test with *Vibrio anguillarum* was conducted at the end of the first trial (nettle extract trial). The nettle and green olive tree trials were conducted sequentially because of the limited number of tanks and the need to preserve a triplicate design for all treatment groups. In addition, after the first trial and its bacterial challenge, the system underwent a disinfection and reconditioning period before the second trial began. To minimize potential temporal effects, each trial included its own control group, fish were obtained from the same supplier, and environmental and husbandry conditions were kept consistent between trials.

#### Extraction of plant extracts and diet preparation

Nettle and green olive tree extracts were obtained with the ethanolic extraction method (Bilen et al. 2011). Briefly, both plants’ leaves were air-dried at room temperature for 2 weeks and ground using a grinder (Yazicilar G1/5L®, Turkey). Then, 40 g of the powder was added into 96% ethanol. The suspension was kept in the dark for 72 h at room temperature and mixed by inverting twice a day. Afterwards, the liquid was evaporated using a rotary evaporator at 80℃, and the remaining extract was re-dissolved in 40 ml of ethanol.

Based on dry matter recovery from the final extract volume, the overall extraction yields were calculated as 6.38% for *P. latifolia* and 5.16% for *U. dioica*.

For feed preparation, the required amount of each extract was diluted with ethanol and applied to the commercial feed by spraying. For each treatment, the final spray volume was adjusted to 100 mL/kg feed. The extract solution was applied gradually in small portions, and the pellets were manually mixed after each spraying step to promote as uniform a distribution as possible. The control diet was treated with the same volume of ethanol without plant extract. After spraying, the prepared feeds were kept in a drying chamber for 72 h to allow ethanol evaporation. The feeds were then stored at + 4 °C for weekly use and at − 20 °C for longer-term storage during the feeding trial. Because the same commercial basal feed was used in all groups and the plant extracts were applied only as low-dose top coatings (50–400 mg/kg feed), the experimental diets were considered iso-nitrogenous and iso-energetic in practical terms. The reported inclusion levels therefore represent nominal target concentrations based on the amount of extract applied per kilogram of feed, as post-coating analytical verification of actual inclusion levels was not performed.

#### Determination of phenolic compounds

Phenolic compounds in both extracts were analyzed with Liquid Chromatography-Tandem Mass Spectrometry (LC–MS/MS). Analyses were performed by Central Research Laboratory of Kastamonu University using a Shimadzu LCMS-8040 instrument.

#### Feeding procedure

The fish were fed the prepared diets for 60 days, twice daily at 9:00 a.m. and 5:00 p.m., to apparent satiation. Feed was offered slowly by hand, and feeding was stopped when the first uneaten pellet was observed in order to minimize feed loss. Therefore, feed consumption for FCR calculations was estimated from the amount of feed offered to each tank. Control groups in both trials received the same feed without any additives. Experimental groups received plant extracts at the rates of 50, 100, 200, and 400 mg per kg feed. Group names were as follows: C1 (control group of the first trial), C2 (control group of the second trial), and X50, X100, X200, and X400, where X refers to either N (nettle) or GOT (green olive tree).

#### Sample collection

To evaluate growth performance, all fish were collected and weighed on days 30 and 60 of the experiments. For hematological, immunological, and gene expression analyses, three fish were sampled from each aquarium on day 60, resulting in nine fish per treatment group. Fish were anesthetized with 0.5 mL/L 2-phenoxyethanol, and blood samples were collected from the caudal vein using sterile syringes. The fish were then euthanized, and spleen and intestine tissues were collected using a dissection kit. The spleen and intestinal samples were transferred to Eppendorf tubes, RNA Later solution (Thermo Fisher Scientific®; Invitrogen) was added, and the samples were stored at −20 °C until use.

The experiments were conducted at the Living Resources Laboratory of the Faculty of Marine Sciences and Technology, Çanakkale Onsekiz Mart University. For each trial, 15 aquaria (100 L each) were used. Seawater was regularly transported from the facility where the fish were obtained, and 20% of the water was renewed daily. A 12 h:12 h light:dark photoperiod was maintained using automatic timers.

#### Growth performance

Herbal additives’ effects on growth performance and feed conversion were evaluated by using the equations below (Hoşsu et al. 2003):

1 $$Weight gain \left(WG, \%\right)=\frac{Final weight \left(g\right)-Initial weight (g)}{Initial weight (g)}\times 100$$

2 $$Specific growth rate \left(SGR, \%/day\right)=\frac{\mathrm{ln}FMW \left(g\right)- \mathrm{ln}IMW (g) }{Number of trial days}\times 100$$

where *FMW* is the final mean weight and *IMW* is the initial mean weight.

3 $$Feed conversion rate \left(FCR\right)=\frac{Feed consumption (g)}{Weight gain (g)}\times 100$$

#### Hematological analyses

Fresh blood samples were used to assess red blood cell (RBC), hemoglobin (HB), and hematocrit (HCT) values with a blood cell count instrument (Mindray/BC 3000® Plus), of which measurement values were previously adjusted for fish blood and verified by Yilmaz (2017). Mean corpuscular volume (MCV), mean corpuscular hemoglobin (MCH), and mean corpuscular hemoglobin concentration (MCHC) were then calculated according to Lewis (2006).

#### Immunological analyses

Respiratory Burst Activity (RBA) was determined from fresh blood samples with slight modifications in the method of Stasiak and Baumann (1996) as described by Yilmaz (2020). Fifty µL blood samples from each fish were placed into 96 plates containing poly-l-lysine. After incubating the samples for one hour, the upper phase was discarded, HBSS was added, and the plates were washed three times. Subsequently, 100 µL of 0.2% NBT was added to each well at and left to incubate for another hour. The cells were fixed with methanol (100%) for 5 min and washed three times with 70% methanol. Sixty µL of 2 M potassium hydroxide and 70 µL of DMSO were transferred to each well of the dried plates. Following these steps, readings were done at a wavelength of 620 nm using a Multiscan spectrophotometer (Thermo Multiscan Go).

Lysozyme (LYS) and myeloperoxidase (MPO) activities were assessed from blood plasma. LYS activity was measured according to Nudo and Catap (2011). Briefly, 10 µL of serum sample was distributed into 96-well plates and diluted by adding 90 µL of HBSS. Thirty-five µL of the diluted solution, containing one 3,3′,5,5′-tetramethylbenzidine dihydrochloride tablet, 2 µL of 30% hydrogen peroxide, and pure water, was added to each well. Before starting the measurement with the spectrophotometer, incubation was performed at 12 ℃ for 2 min, and at the end of this time, 35 µL of sulfuric acid was added to the wells to stop the reaction. Spectrophotometric readings were then performed (OD: 450 nm). In the present study, lysozyme values are expressed as µg/mL, in accordance with the output format of the assay.

MPO was assayed as modified by Yilmaz (2020) from Kumari and Sahoo (2006) and Quade and Roth (1997). Lyophilized *Micrococcus luteus* was weighed (0.075 g) and dissolved in a buffer mixture (pH 5.8) prepared using 100 mL of Na_2_HPO_4_–2H_2_O and citric acid. Twenty-five µL of serum sample was placed in 96-well plates in triplicate, and 175 µL of *M. luteus* suspension was added. Immediately following these steps, kinetic measurements were initiated at 450 nm using the spectrophotometer.

#### Expression analysis of cytokines

##### RNA isolation

RNA isolation was performed from the collected spleen and intestine samples on the 30th and 60th days. Tissue samples were homogenized in 500 µL Trizol (RiboEx, GeneAll®), then centrifuged at 13,000 rpm. Then, 200 µL chloroform was added to the supernatant, 200 µL chloroform was added. After repeating this process, the supernatant was transferred to a new vial, and 500 µL isopropanol was added. The suspension was gently mixed and incubated at −30℃ for 7 min. It was again centrifuged at 13,000 rpm, and the precipitate was rinsed with 70% ethanol. After a final centrifuge step, samples were incubated in a biosafety cabinet to evaporate the alcohol. Twenty µL of nuclease-free water (NFW) was added on top, and the suspension was incubated on ice to obtain the RNA samples. Purity and concentrations of the RNA samples were confirmed by determining absorbance values at 260/280 nm with a spectrophotometer (Thermo Fisher Scientific®, Multiskan GO). Finally, the samples were diluted to 20 ng/µL with NFW and stored at − 80℃ until cDNA synthesis.

##### cDNA synthesis

Complementary DNA was synthesized from the isolated RNA samples using A.B.T.™ cDNA Synthesis Kit with RNase Inh. High Capacity (Atlas Biotechnology, Ankara, Turkey; Cat. No. C03-01–05) according to the manufacturer’s instructions. The reverse transcription program consisted of one cycle at 25 °C for 10 min, 37 °C for 120 min, and 85 °C for 5 min. The purity and concentration of the synthesized cDNA were determined as described in the RNA isolation section. The cDNA samples were then diluted to 20 ng/µL with nuclease-free water (NFW).

##### RT-qPCR procedures

This study investigated the expression levels of *β-actin*, *IL-1β*, *IL-6*, *IL-10*, *IL-18*, and *TNF-α* genes. RT-qPCR reactions were carried out in a final volume of 20 µL using 10 µL of A.B.T.™ 2X qPCR Mastermix (Atlas Biotechnology, Ankara, Turkey), which contains SYBR Green and ROX, 2 µL of 10 µM forward and reverse primers (Table 2), 2 µL of template cDNA, and 4 µL of nuclease-free water. Amplification was performed in an Applied Biosystems® StepOnePlus™ Real-Time PCR System, with an initial denaturation at 95 °C for 10 min, followed by 40 cycles of denaturation at 95 °C for 15–30 s, annealing at primer-specific temperatures ranging from 55 to 60 °C for 30 s, and extension at 72 °C for 30 s.

**Table 2:** Primer pairs used in the study

Gene	Primer (5′−3′)	Source
*β-Actin*	F5′ TCTGTCTGGATCGGAGGCTC 3′ R5′ AAGCATTTGCGGTGGACG 3′	Dominguez et al. (2021)
*IL-1β*	F5′ AGCGACATGGCACGATTTC 3′ R5′ GCACTCTCCTGGCACATATCC 3′	Montero et al. (2010)
*IL-6*	F5′ TGCTTTCACCCCTACAGACG 3′ R5′ GCTTCAAACTCCTGCCTGTGG 3′	Güroy et al. (2022)
*IL-10*	F5′ CAGTCTTTCCTCTGCACCGT 3′ R5′ CAGGCGAACGCTGTTTTGAA 3′	Güroy et al. (2022)
*IL-18*	F5′ GGAACACAGCCTGAATGCAAA 3′ R5′ AGTCCGGTAGAACACAGCAC 3′	^a^ Gene no (NCBI code): JX976626.1
*TNF-α*	F: 5′ CTCACACCTCTCAGCCACAG 3′ R: 5′ TTCCGTCTCCAGTTTGTCG 3′	Mathlouthi (2008)

To verify amplification specificity, a melt curve analysis was performed at the end of each run between 65 °C and 95 °C. A single sharp peak was obtained for each target gene, indicating the absence of detectable primer-dimer formation and nonspecific amplification. Relative gene expression levels were calculated using the 2^−ΔΔCt^ method (Altunoglu et al. 2017), with *β-actin* used as the reference gene.

#### Challenge test

At the end of the first trial (nettle supplementation), fish were challenged by intraperitoneal injection with 100 µL of a *Vibrio* (*Listonella*) *anguillarum* suspension previously isolated from diseased fish. The final challenge dose was 1 × 10^7^ CFU/fish. The bacterial strain was reactivated in Tryptic Soy Broth supplemented with 1.5% NaCl and incubated at 25 °C for 24 h. Bacterial cells were then collected by centrifugation (3000 rpm, 10 min), washed twice with sterile physiological saline/PBS, and adjusted spectrophotometrically at 600 nm according to McFarland standards. Final bacterial concentration was verified by serial dilution and plate counting on Tryptic Soy Agar supplemented with 1.5% NaCl.

After injection, mortalities were recorded daily for 10 days, and survival rates were calculated according to the following formula (Rairakhwada et al. 2007).

$$Survival Rate \left(SR, \%\right)=\frac{Number of survived fish}{Number of infected fish}x100$$

The challenge dose was selected based on published pathogenicity studies in gilthead seabream (Balebona et al. 1998; Bakopoulos et al. 2018), which indicate that this inoculum level provides sufficient pathogen pressure for evaluating dietary immunostimulants. Infection was confirmed by re-isolation of *V. anguillarum* from diseased fish using conventional microbiological methods.

A challenge test was also planned for the green olive tree trial. However, in that trial, the expected mortality was not achieved in the control group because the *V. anguillarum* strain showed reduced virulence during laboratory passage. Therefore, those survival data were considered unsuitable for efficacy evaluation and were not included in the manuscript.

#### Statistical analyses

Statistical analyses were performed using IBM SPSS Statistics 23.0 (IBM Corp., Armonk, NY, USA). Data are expressed as mean ± standard error (SE). The assumptions of normality and homogeneity of variance were assessed using the Shapiro–Wilk and Levene’s tests, respectively. Following confirmation of these assumptions, one-way ANOVA was performed, with Duncan’s multiple range test used for data with homogeneous variances and Tamhane’s T2 test applied when homogeneity of variance was not met. For growth performance, hematological parameters, and nonspecific immune responses, the tank was treated as the experimental unit, and values are presented as mean ± SE of three replicate tanks per group. For gene expression analyses, tissue samples were obtained from nine fish per treatment group, comprising three fish sampled from each of the three replicate tanks. Ct values of the reference gene (*β-actin*) were compared among tanks, and no significant tank effect was detected (*p* > 0.05). Therefore, the nine individual fish were considered biological replicates for molecular analyses. Since the nettle and green olive tree extract experiments were conducted as separate trials at different times, with different fish populations and independent control groups, the datasets were analyzed separately rather than within a unified factorial framework.

## Results

Physicochemical water quality parameters measured during both trials are given in Table 3.

**Table 3:** Ranges of physicochemical water-quality parameters measured

	**Temperature (℃)**	**Salinity (ppt)**	**Dissolved oxygen (mg/L)**	**pH**	**Total ammonia (mg/L)**	**Nitrite (mg/L)**	**Nitrate (mg/L)**
1 st trial (N)	16–20	35–36	5.5–6.5	7.5–8.5	0.01–0.05	0.01–0.03	0.8–1.1
2nd trial (GOT)	20–23	33–34	5–6	7.5–8.5	0.02–0.07	0.03–0.05	0.8–1.1

### Phenolic compounds in the extracts

The LC–MS/MS results of both extracts are presented in Table 4. It was found that nettle (N) extract contained 43,625 µg/kg rutin trihydrate, 10,997 µg/kg catechin, 776 µg/kg caffeic acid, 194 µg/kg trans-ferulic acid, 156 µg/kg ellagic acid, 128 µg/kg quercetin, and 75 µg/kg cinnamic acid. On the other hand, green olive tree (GOT) extract composed of 3120 µg/kg rutin trihydrate, 197 µg/kg luteolin, 63 µg/kg cinnamic acid, and 44 µg/kg quercetin.

**Table 4:** Phenolic compounds detected in the nettle and green olive tree extracts (µg/kg)

Compound	Nettle extract	Green olive tree extract
Catechin	10,997	n.d
Ellagic acid	156	n.d
Rutin trihydrate	43,625	3120
Quercetin	128	44
Luteolin	n.d	197
Cinnamic acid	75	63
Caffeic acid	776	n.d
Trans ferulic acid	194	n.d

*n.d.* not detected

### Growth performance

Growth performance parameters are presented in Table 5. According to the results, N extract supplementation did not affect growth performance in the fish on the 30th and 60th days (*p* > 0.05). On the other hand, GOT extract-supplemented groups showed significantly lower feed conversion rate (FCR) values on the 30th day compared to the control group (*p* < 0.05). On the 60th day, however, none of the investigated parameters was different among groups (*p* > 0.05).

**Table 5:** Growth performance parameters of *Sparus aurata* individuals after 30 and 60 days of supplementation with nettle or green olive tree extracts

	**Trial groups (30 days)**				
	**C1**	**N50**	**N100**	**N200**	**N400**
Initial weight (g)	1.06 ± 0.10	1.09 ± 0.09	1.07 ± 0.08	1.09 ± 0.07	1.08 ± 0.07
Final weight (g)	3.88 ± 0.11	3.69 ± 0.27	3.64 ± 0.27	3.93 ± 0.43	3.92 ± 0.47
Weight gain (%)	265.33 ± 2.42	239.87 ± 4.18	241.26 ± 6.54	260.06 ± 12.44	262.93 ± 4.24
FCR	1.37 ± 0.01	1.37 ± 0.01	1.35 ± 0.02	1.33 ± 0.01	1.35 ± 0.02
SGR (% day^−1^)	4.32 ± 0.02	4.08 ± 0.04	4.09 ± 0.06	4.26 ± 0.12	4.30 ± 0.04
	**Trial groups (60 days)**				
	**C1**	**N50**	**N100**	**N200**	**N400**
Initial weight (g)	1.06 ± 0.10	1.09 ± 0.09	1.07 ± 0.08	1.09 ± 0.07	1.08 ± 0.07
Final weight (g)	8.97 ± 0.87	8.63 ± 0.90	9.31 ± 0.83	9.06 ± 0.87	8.14 ± 0.91
Weight gain (%)	744.09 ± 19.02	693.19 ± 16.43	770.93 ± 14.78	731.72 ± 67.30	653.14 ± 42.91
FCR	1.37 ± 0.01	1.38 ± 0.01	1.22 ± 0.02	1.26 ± 0.06	1.30 ± 0.03
SGR (% day^−1^)	3.33 ± 0.03	3.15 ± 0.03	3.30 ± 0.03	3.21 ± 0.13	3.05 ± 0.09
	**Trial groups (30 days)**				
	**C2**	**GOT50**	**GOT100**	**GOT200**	**GOT400**
Initial weight (g)	3.24 ± 0.45	3.30 ± 0.49	3.42 ± 0.45	3.13 ± 0.42	3.31 ± 0.55
Final weight (g)	8.87 ± 1.21	9.14 ± 1.19	9.36 ± 1.08	9.30 ± 0.98	8.92 ± 1.34
Weight gain (%)	173.49 ± 1.96	177.21 ± 8.10	173.70 ± 12.71	198.26 ± 7.79	170.57 ± 7.16
FCR	1.40 ± 0.03^a^	1.35 ± 0.03^ab^	1.25 ± 0.03^b^	1.24 ± 0.02^b^	1.37 ± 0.02^a^
SGR (% day^−1^)	3.26 ± 0.10	3.30 ± 0.06	3.30 ± 0.13	3.50 ± 0.12	3.27 ± 0.12
	**Trial groups (60 days)**				
	**C2**	**GOT50**	**GOT100**	**GOT200**	**GOT400**
Initial weight (g)	3.24 ± 0.45	3.30 ± 0.49	3.42 ± 0.45	3.13 ± 0.42	3.31 ± 0.55
Final weight (g)	14.95 ± 1.46	14.82 ± 1.68	15.23 ± 1.93	15.07 ± 1.36	14.71 ± 1.79
Weight gain (%)	360.51 ± 2.08	349.60 ± 16.88	339.50 ± 16.96	384.52 ± 26.68	346.14 ± 15.90
FCR	1.35 ± 0.05	1.39 ± 0.04	1.30 ± 0.03	1.31 ± 0.06	1.40 ± 0.05
SGR (% day^−1^)	2.24 ± 0.01	2.20 ± 0.06	2.16 ± 0.06	2.32 ± 0.09	2.19 ± 0.06

*n* = 9; mean ± SE. Differences between groups with different exponential letters within the same period and trial are statistically significant (*p* < 0.05)

*FCR* feed conversion rate, *SGR* specific growth rate, *C1* first trial’s control group, *C2* second trial’s control group, *N50, N100, N200, N400* Nettle extract-supplemented groups at 50, 100, 200, and 400 mg/kg feed, respectively; *GOT50, GOT100, GOT200, GOT400* green olive tree extract-supplemented groups at 50, 100, 200, and 400 mg/kg feed, respectively

### Hematological parameters

Blood parameters assessed from the fish on the 60th day of the experiments are displayed in Table 6. The results showed that blood parameters in the N groups were similar to those of the control (*p* > 0.05). In contrast, although GOT-supplemented groups also did not show significantly different RBC, HB, and MCH values (*p* > 0.05), the GOT200 group exhibited higher HCT and MCV values than the control (*p* < 0.05). Furthermore, the MCHC values of the GOT100, GOT200, and GOT400 groups were lower than those of the control (*p* < 0.05).

**Table 6:** Blood parameters of *Sparus aurata* individuals after 60 days of supplementation with nettle or green olive tree extracts

Nettle	RBC (10^6^/mm^3^)	HB (g/dL)	HCT (%)	MCV (μm^3^)	MCH (pg)	MCHC (%)
C1	2.90 ± 0.11	6.94 ± 0.33	28.29 ± 1.34	97.62 ± 1.82	23.93 ± 0.28	24.54 ± 0.46
N50	2.90 ± 0.11	6.86 ± 0.33	30.41 ± 1.76	104.60 ± 2.40	23.62 ± 0.20	22.61 ± 0.45
N100	2.64 ± 0.13	6.05 ± 0.16	25.12 ± 0.51	95.95 ± 5.51	23.00 ± 0.54	24.12 ± 0.90
N200	2.79 ± 0.27	5.90 ± 0.40	27.15 ± 2.51	97.58 ± 2.66	21.34 ± 0.76	21.88 ± 0.55
N400	2.86 ± 0.08	6.68 ± 0.17	27.07 ± 1.95	94.37 ± 4.50	23.37 ± 0.34	24.91 ± 1.12
Green olive tree	**RBC (10**^**6**^**/mm**^**3**^**)**	**HB (g/dL)**	**HCT (%)**	**MCV (μm**^**3**^**)**	**MCH (pg)**	**MCHC (%)**
C2	3.80 ± 0.22	8.12 ± 0.24	24.63 ± 0.89^b^	65.10 ± 1.74^c^	21.59 ± 1.45	33.06 ± 1.30^a^
GOT50	3.64 ± 0.20	7.87 ± 0.36	26.04 ± 2.04^ab^	71.36 ± 1.72^bc^	21.67 ± 0.20	30.43 ± 0.97^ab^
GOT100	3.71 ± 0.07	7.64 ± 0.13	27.17 ± 0.65^ab^	73.21 ± 0.34^ab^	20.61 ± 0.06	28.15 ± 0.21^b^
GOT200	3.74 ± 0.19	7.97 ± 0.31	29.42 ± 0.51^a^	79.10 ± 2.70^a^	21.35 ± 0.34	27.06 ± 0.70^b^
GOT400	3.59 ± 0.11	7.74 ± 0.18	28.19 ± 0.19^ab^	78.65 ± 1.76^ab^	21.58 ± 0.14	27.46 ± 0.46^b^

*n* = 9; mean ± SE. Differences between groups having different exponential letters in the same trial are statistically significant (*p* < 0.05)

*RBC* red blood cell, *HB* hemoglobin, *HCT* hematocrit, *MCV* mean corpuscular volume, *MCH* mean corpuscular hemoglobin, *MCHC* mean corpuscular hemoglobin concentration, *C1* first trial’s control group, *C2* second trial’s control group, *N50, N100, N200, N400* nettle extract-supplemented groups at 50, 100, 200, and 400 mg/kg feed, respectively; *GOT50, GOT100, GOT200, GOT400* green olive tree extract-supplemented groups at 50, 100, 200, and 400 mg/kg feed, respectively

### Immunological findings

**Fig. 2:**
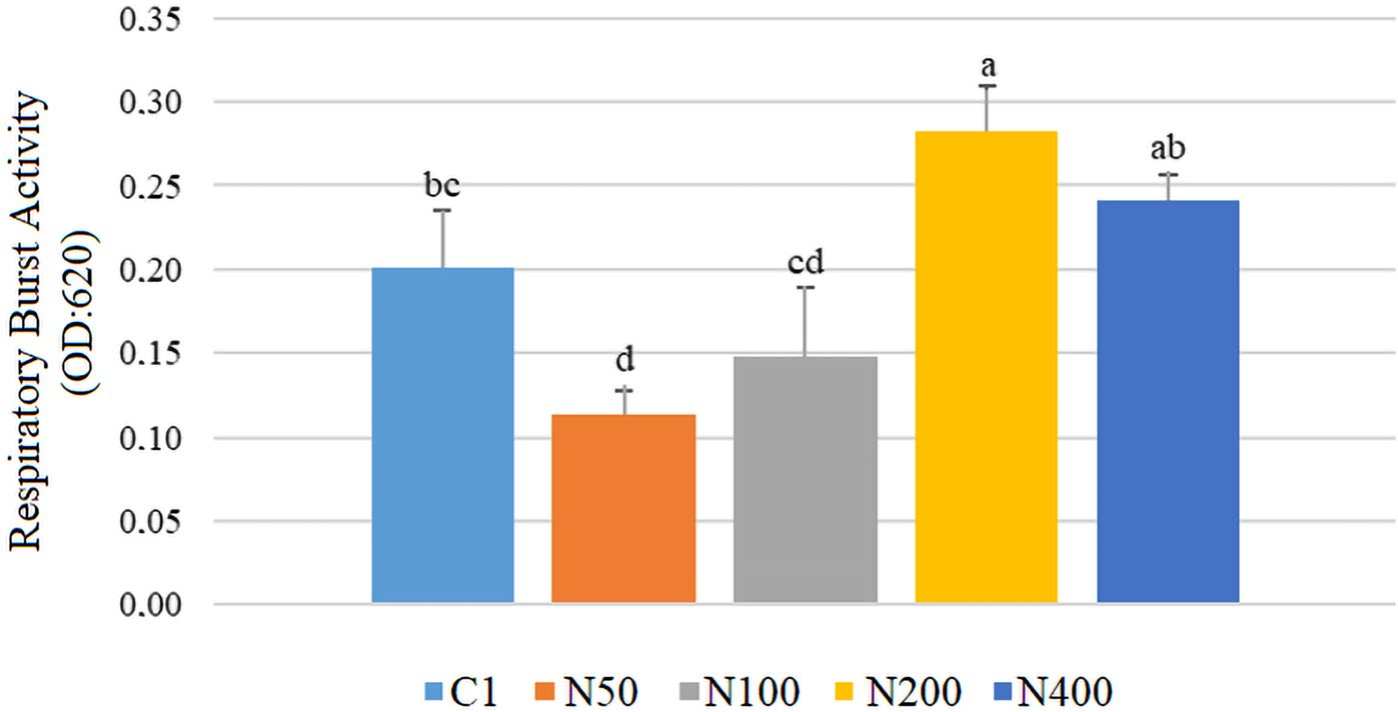
Respiratory burst activity (RBA) of *Sparus aurata* individuals after 60 days of supplementation with nettle extract (Different letters on bars indicate statistically significant differences between groups (*n* = 9, *p* < 0.05). Data are presented as mean ± SE. C1: first trial’s control group; N50, N100, N200, and N400: nettle extract-supplemented groups at 50, 100, 200, and 400 mg/kg feed, respectively)

Figure 2 shows the results of respiratory burst activity (RBA). After N supplementation, the highest RBA was observed in the N200 group (0.28), while the lowest was in the N50 group (0.11). RBA values of the control, N100, and N400 were 0.20, 0.15, and 0.24, respectively. In this study, we were unable to perform RBA analysis in the GOT-supplemented fish. This was discussed at the end of the discussion section.

**Fig. 3:**
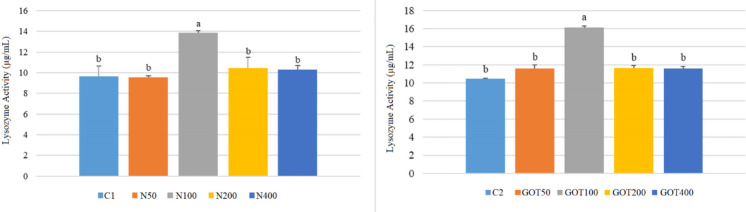
Lysozyme (LYS) activity of *Sparus aurata* individuals after 60 days of supplementation with nettle or green olive tree extracts (different letters on bars indicate statistically significant differences between groups (*n* = 9, *p* < 0.05). Data are presented as mean ± SE. C1: first trial’s control group; C2: second trial’s control group; N50, N100, N200, and N400: nettle extract-supplemented groups at 50, 100, 200, and 400 mg/kg feed, respectively; GOT50, GOT100, GOT200, and GOT400: green olive tree extract-supplemented groups at 50, 100, 200, and 400 mg/kg feed, respectively)

LYS activity results are given in Fig. 3. The highest LYS in the first trial was in the N100 group with 13.84 µg/mL. The remaining groups showed similar LYS values, ranging from 9.56 to 10.96 µg/mL Similarly, in the second trial, the GOT100 group showed the highest LYS at 16.10 µg/mL Likewise, all other groups including the control had similar values ranging between 10.45 and 11.63 µg/mL.

**Fig. 4:**
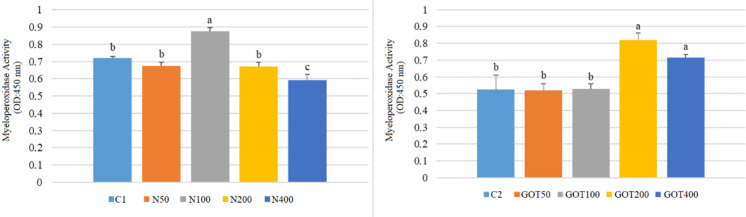
Myeloperoxidase (MPO) activity of *Sparus aurata* individuals after 60 days of supplementation with nettle or green olive tree extracts (different letters on bars indicate statistically significant differences between groups (*n* = 9, *p* < 0.05). Data are presented as mean ± SE. C1: first trial’s control group; C2: second trial’s control group; N50, N100, N200, and N400: nettle extract-supplemented groups at 50, 100, 200 and 400 mg/kg feed, respectively; GOT50, GOT100, GOT200, and GOT400: green olive tree extract-supplemented groups at 50, 100, 200, and 400 mg/kg feed, respectively)

Figure 4 shows MPO activity results. According to the analysis, the N100 group exhibited the highest MPO activity (0.87) in the first trial. Moreover, the N400 group’s MPO activity was the lowest at 0.59. MPO values of the control, N50 and N200 groups were 0.72, 0.67, and 0.67, respectively. In the second trial, the highest MPO was detected in the GOT200 group (0.82), followed by the GOT400 group (0.72). Other groups had similar MPO activities ranging from 0.51 to 0.52.

### Gene expression levels

**Fig. 5:**
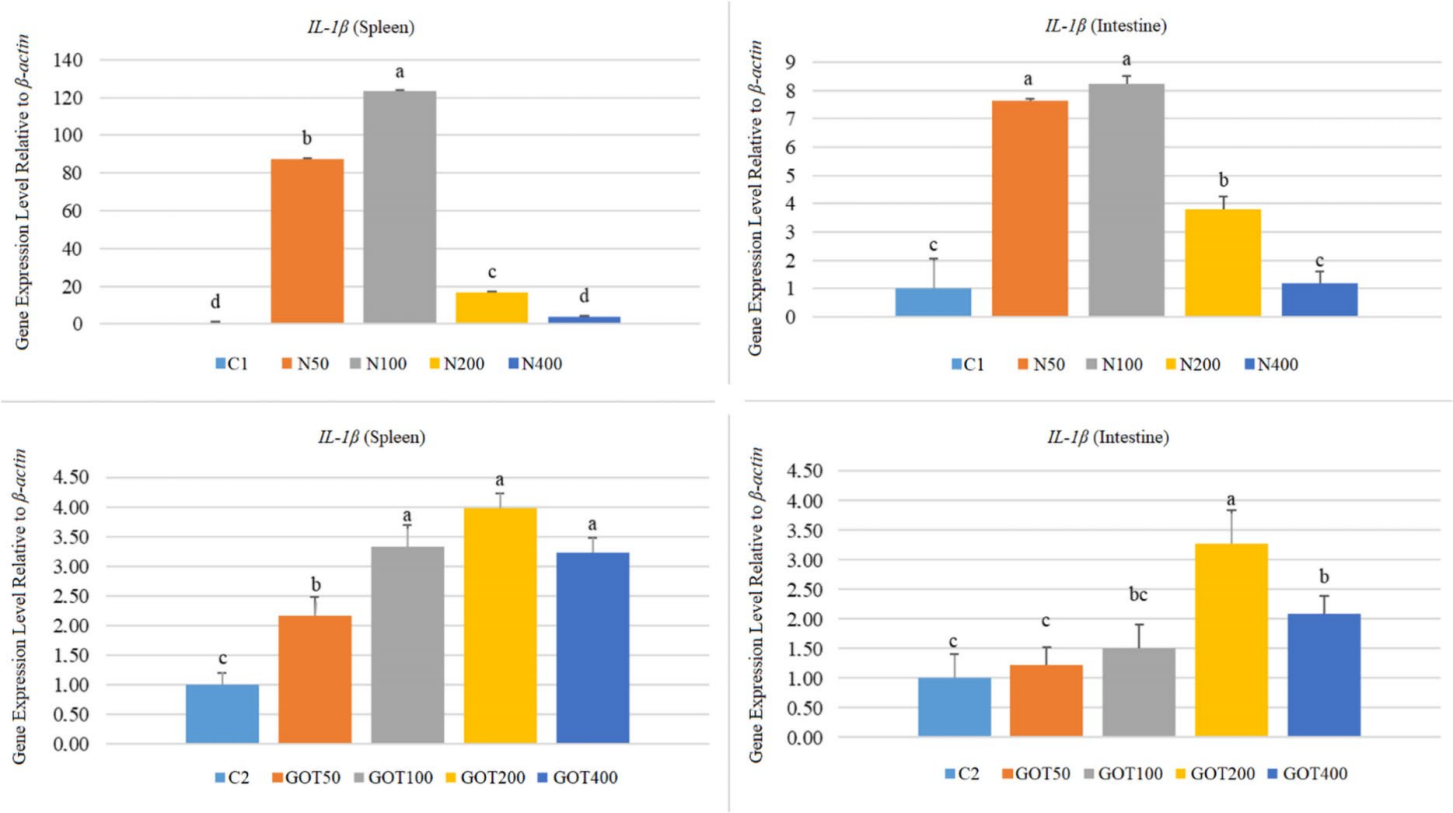
*IL-1β* gene expression levels of *Sparus aurata* individuals after 60 days of supplementation with nettle or green olive tree extracts (Different letters on bars indicate statistically significant differences between groups (*n* = 9, *p* < 0.05). Data are presented as mean ± SE. C1: first trial’s control group; C2: second trial’s control group; N50, N100, N200, and N400: nettle extract-supplemented groups at 50, 100, 200, and 400 mg/kg feed, respectively; GOT50, GOT100, GOT200, and GOT400: green olive tree extract-supplemented groups at 50, 100, 200, and 400 mg/kg feed, respectively)

*IL-1β* gene expression results are displayed in Fig. 5. After N supplementation, spleen *IL-1β* gene expression in the N50, N100 and N200 groups increased (*p* < 0.05) 87.58, 123.28, and 16.97-fold, respectively. Similarly, intestine *IL-1β* expression increased (*p* < 0.05) 7.64, 8.25, and 3.81-fold, respectively. The group supplemented with 400 mg/kg N extract showed slightly increased (*p* > 0.05) *IL-1β* expression in both organs. Likewise, GOT-supplemented groups had higher expression in both organs. Spleen expression was increased (*p* < 0.05) 2.16, 3.32, 3.98, and 3.24-fold following 50, 100, 200, and 400 mg/kg GOT supplementation, respectively. Intestine *IL-1β* expression in the GOT200 and GOT400 groups increased (*p* < 0.05) 3.25- and 2.08-fold, respectively. On the other hand, intestine *IL-1β* expression levels were not affected (*p* > 0.05) following 50 or 100 mg/kg GOT supplementation.

**Fig. 6:**
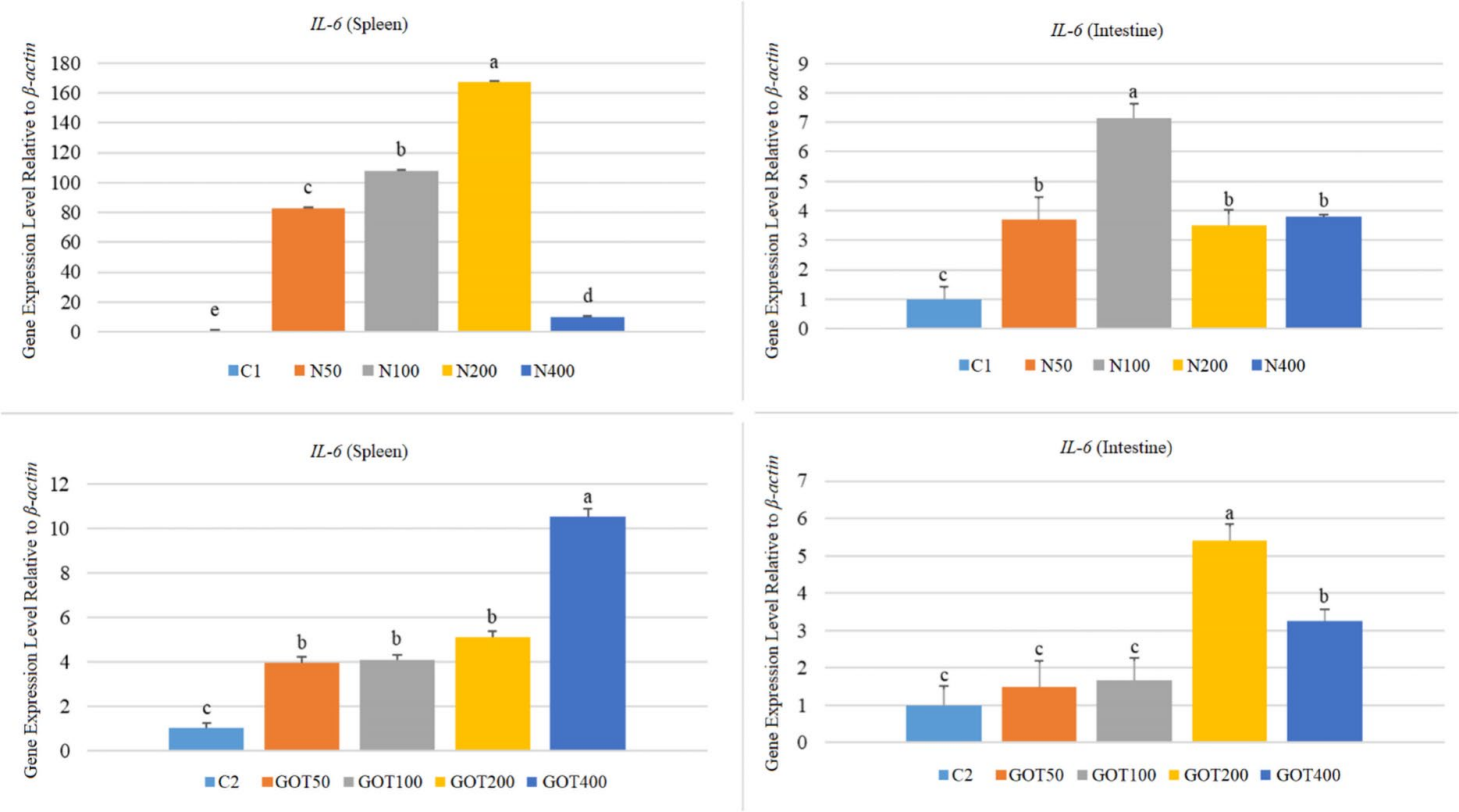
*IL-6* gene expression levels of *Sparus aurata* individuals after 60 days of supplementation with nettle or green olive tree extracts (different letters on bars indicate statistically significant differences between groups (*n* = 9, *p* < 0.05). Data are presented as mean ± SE. C1: first trial’s control group; C2: second trial’s control group; N50, N100, N200, and N400: nettle extract-supplemented groups at 50, 100, 200, and 400 mg/kg feed, respectively; GOT50, GOT100, GOT200, and GOT400: green olive tree extract-supplemented groups at 50, 100, 200, and 400 mg/kg feed, respectively)

All groups in the first trial showed increased (*p* < 0.05) *IL-6* gene expression levels in both organs (Fig. 6). The highest *IL-6* expression in the spleen was in the N200 group (167.38-fold), whereas the highest in the intestine was in the N100 group (7.15-fold). Similarly, GOT supplementation caused elevated (*p* < 0.05) *IL-6* gene expression levels in the spleen, with the highest at 10.52-fold in the GOT400 group. Moreover, intestine expressions were the highest (*p* < 0.05) in the GOT200 group (5.39-fold). The intestine of the GOT400 group also showed an increase (*p* < 0.05) in the *IL-6* gene expression (3.26-fold). Furthermore, although slightly increased (*p* > 0.05), the GOT50 and GOT100 groups had similar intestine *IL-6* gene expression levels to that of the control group.

**Fig. 7:**
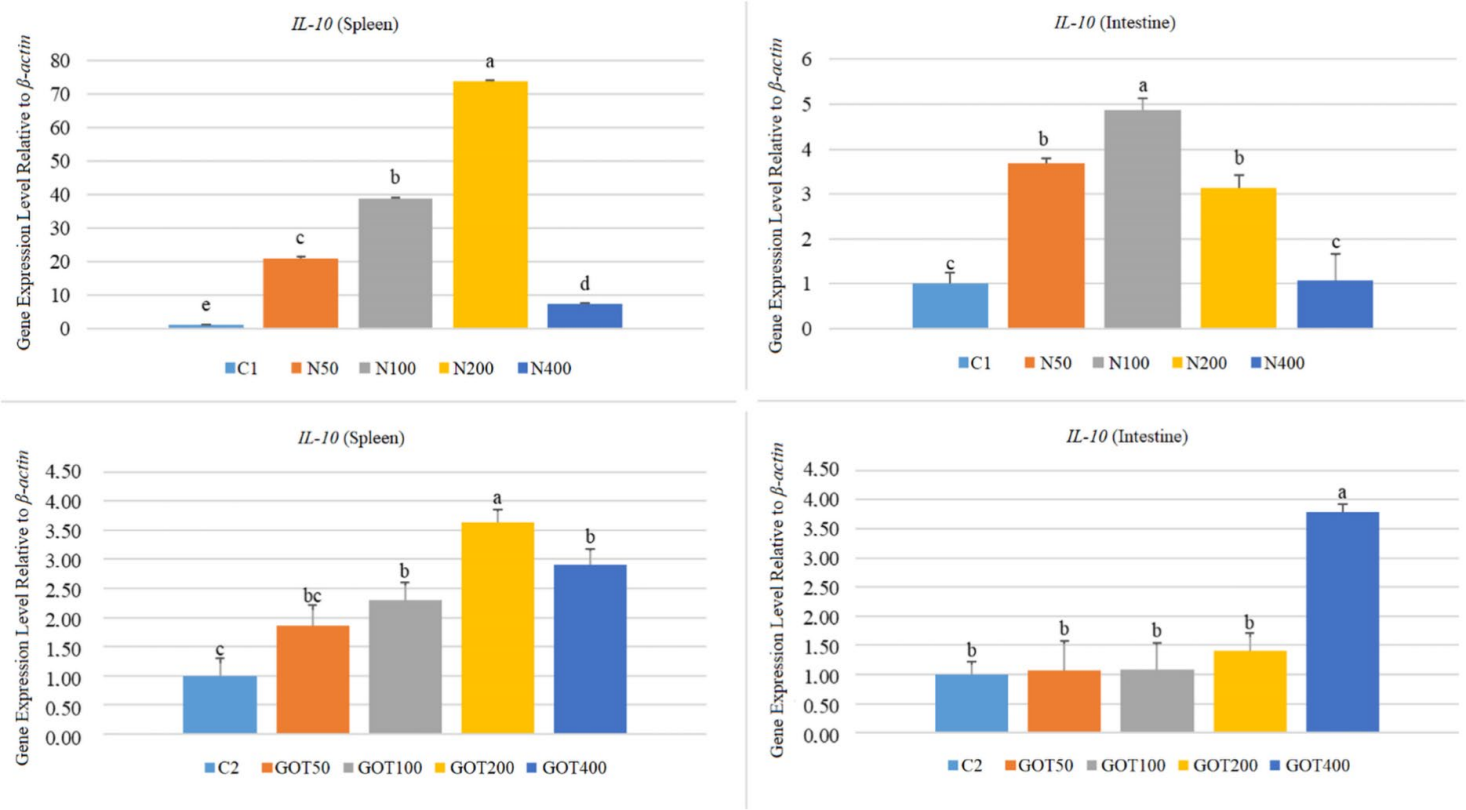
*IL-10* gene expression levels of *Sparus aurata* individuals after 60 days of supplementation with nettle or green olive tree extracts (different letters on bars indicate statistically significant differences between groups (*n* = 9, *p* < 0.05). Data are presented as mean ± SE. C1: first trial’s control group; C2: second trial’s control group; N50, N100, N200, and N400: nettle extract-supplemented groups at 50, 100, 200, and 400 mg/kg feed, respectively; GOT50, GOT100, GOT200, and GOT400: green olive tree extract-supplemented groups at 50, 100, 200, and 400 mg/kg feed, respectively)

Figure 7 shows *IL-10* gene expression results. Again, *IL-10* gene expression levels increased (*p* < 0.05) in all experimental groups in the first trial, except in the intestine of the N400 group, which, although increased slightly, did not differ from the control group significantly (*p* > 0.05). The highest increase in the spleen was in the 200 mg/kg N-supplemented group (73.90-fold), while it was in the intestine in the 100 mg/kg-supplemented group (4.88-fold). After GOT supplementation, *IL-10* gene expression increased (*p* < 0.05) in the spleens of the GOT100, GOT200, and GOT400 groups (2.30, 3.64, and 2.90-fold, respectively). In contrast, it increased (*p* < 0.05) in the intestine only in the GOT400 group (3.78-fold). Furthermore, *IL-10* expression in the spleen of the GOT50 group and in the intestines of the GOT50, GOT100 and GOT200 groups elevated slightly (*p* > 0.05) but not significantly.

**Fig. 8:**
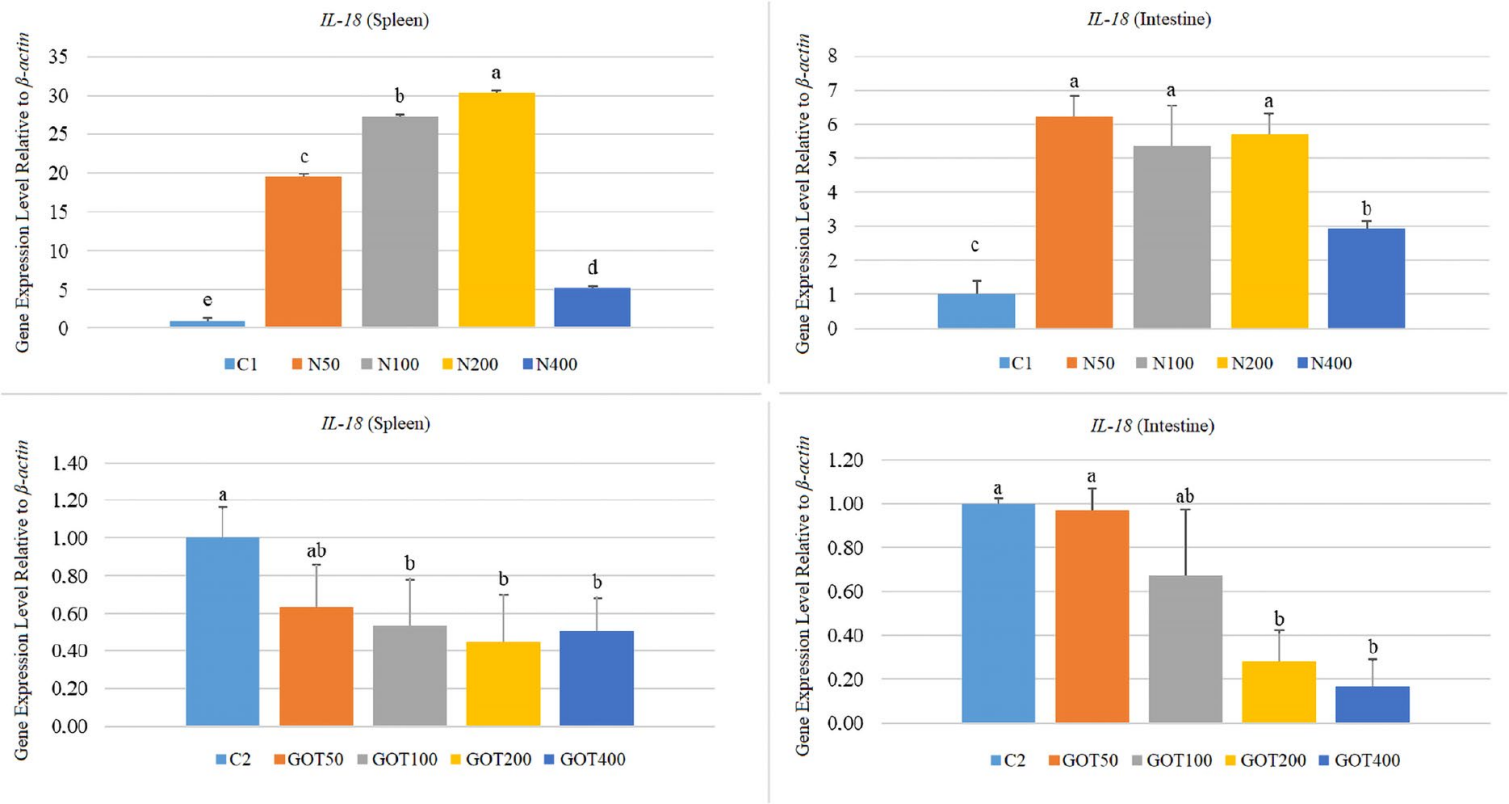
*IL-18* gene expression levels of *Sparus aurata* individuals after 60 days of supplementation with nettle or green olive tree extracts (different letters on bars indicate statistically significant differences between groups (*n* = 9, *p* < 0.05). Data are presented as mean ± SE. C1: first trial’s control group; C2: second trial’s control group; N50, N100, N200, and N400: nettle extract-supplemented groups at 50, 100, 200, and 400 mg/kg feed, respectively; GOT50, GOT100, GOT200, and GOT400: green olive tree extract-supplemented groups at 50, 100, 200, and 400 mg/kg feed, respectively)

*IL-18* gene expression levels increased (*p* < 0.05) in both organs of the N-supplemented groups (Fig. 8). The highest increase was at 30.28-fold in the N200 group for the spleen and at 19.48, 27.22 and 5.15-fold in the N50, N100 and N200 groups for the intestine, respectively. Conversely, GOT supplementation caused decreased (*p* < 0.05) *IL-18* gene expression in the spleens of the GOT100 (0.53-fold), GOT200 (0.45-fold), and GOT400 (0.51-fold) groups. Similar to the spleen expression levels, intestine *IL-18* expressions were lower (*p* < 0.05) in the GOT200 (0.28-fold) and GOT400 (0.17-fold) groups. The remaining groups were not affected (*p* > 0.05) by the supplementation.

**Fig. 9:**
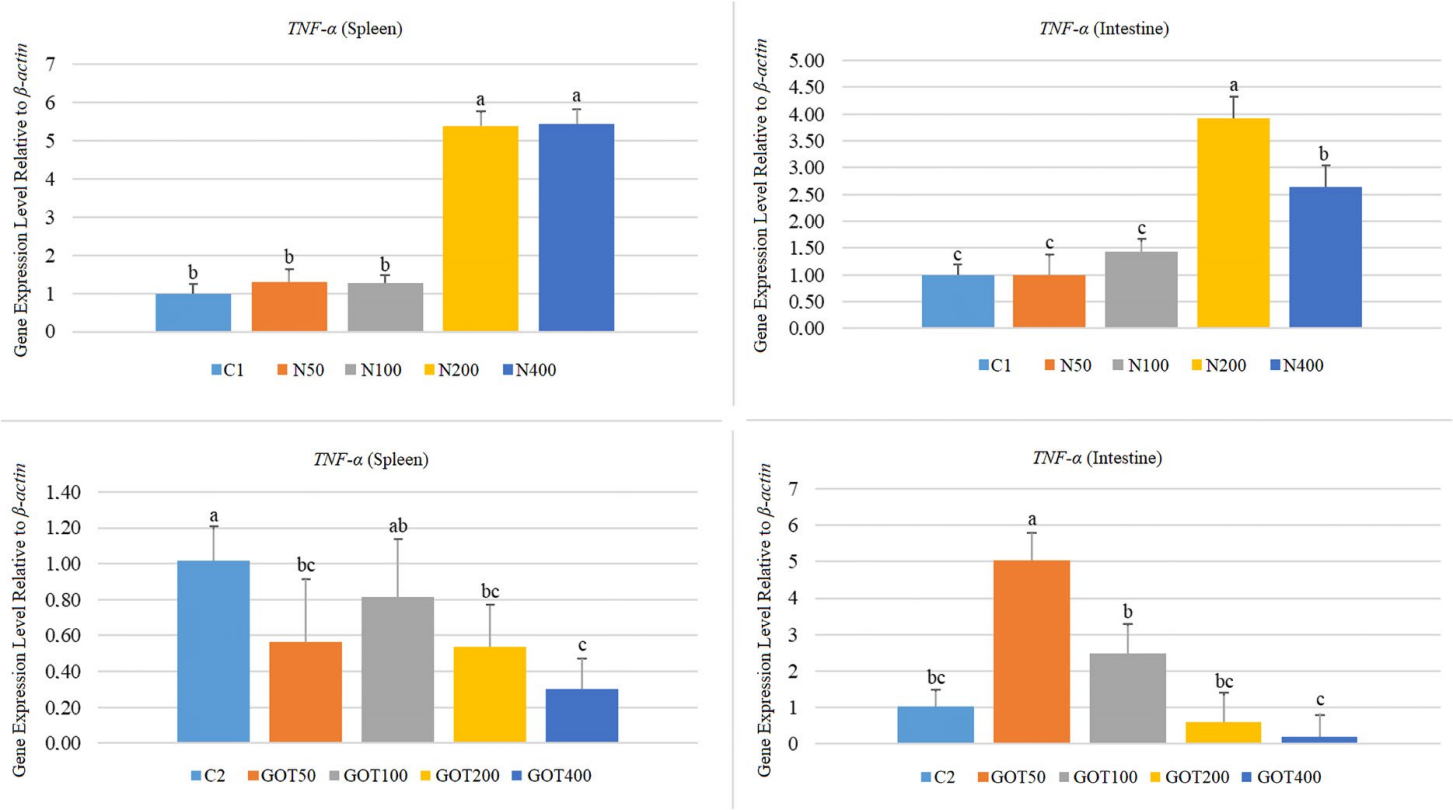
*TNF-α* gene expression levels of *Sparus aurata* individuals after 60 days of supplementation with nettle or green olive tree extracts (different letters on bars indicate statistically significant differences between groups (*n* = 9, *p* < 0.05). Data are presented as mean ± SE. C1: first trial’s control group; C2: second trial’s control group; N50, N100, N200, and N400: nettle extract-supplemented groups at 50, 100, 200, and 400 mg/kg feed, respectively; GOT50, GOT100, GOT200, and GOT400: green olive tree extract-supplemented groups at 50, 100, 200, and 400 mg/kg feed, respectively)

Both organs in the N-supplemented groups showed a similar trend regarding *TNF-α* gene expression levels (Fig. 9). *TNF-α* expression increased (*p* < 0.05) in the N200 and N400 groups in both organs (5.39 and 5.45-fold in the spleen; 3.93 and 2.64-fold in the intestine, respectively). On the other hand, expression levels in the spleens and intestines of the N50 and N100 groups were similar (*p* > 0.05) to those of their respective control groups. In contrast, GOT supplementation caused decreased *TNF-α* expression in the spleen. Expression levels in the GOT50, GOT200, and GOT400 decreased (*p* < 0.05) 0.56, 0.54, and 0.30-fold, respectively. On the contrary, intestine *TNF-α* expression increased (*p* < 0.05) 5.04-fold after 50 mg/kg GOT supplementation. Although slightly varied (*p* > 0.05), other groups (in the spleen of the GOT100, and in the intestines of the GOT100, GOT200, and GOT400 groups) displayed similar *TNF-α* expression levels to their respective control groups.

### Challenge test results

**Fig. 10:**
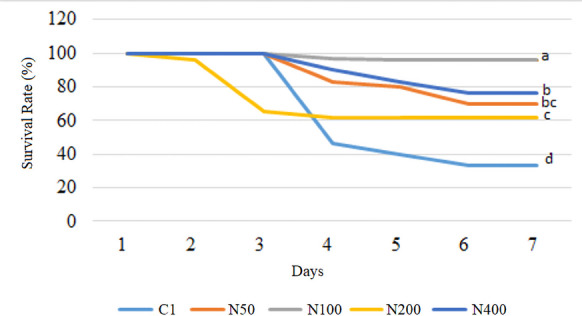
Survival rates of *Sparus aurata* individuals after 60 days of supplementation with nettle extract and subsequent *Vibrio* (*Listonella*) *anguillarum* infection (C1: first trial’s control group; N50, N100, N200, and N400: Nettle extract-supplemented groups at 50, 100, 200, and 400 mg/kg feed, respectively)

After the feeding trial was completed, N-supplemented groups were infected with *Vibrio* (*Listonella*) *anguillarum* to assess the effect of nettle extract supplementation on disease resistance. As a result of the challenge test, the highest survival rate (96%) was observed in the N100 group (*p* < 0.05). Moreover, all other experimental groups showed significantly higher survival rates than the control (p < 0.05). Survival rates were 33%, 70%, 96%, 62%, and 76% in the control, N50, N100, N200 and N400 groups, respectively (Fig. 10). In this study, we were unable to carry out a challenge test in the GOT-supplemented fish. This was discussed at the end of the discussion section.

## Discussion

In fish farming, the use of immunostimulants, which have potential for disease control as an alternative to drugs, chemicals, and antibiotics, is attracting the attention of many researchers today. In this context, the focus has generally been on the use of medicinal plant products to modulate the immune response, take preventive measures, and, especially, control fish diseases (Awad and Austin 2010; Bilen et al. 2021; Secombes 1996; Terzi et al. 2021). As a result of testing different herbal extracts on various fish species, many studies with overlapping or independent results have contributed to the literature in the fields of growth performance, hematology, and innate and adaptive immunity (Altunoglu et al. 2017; Baba 2017; Bilen et al. 2011; Çelik 2020; Sönmez et al. 2022; Yilmaz et al. 2020). Growing evidence suggests that medicinal plants used as feed additives in fish may enhance growth and positively influence immunity. Phenolic compounds present in the plants often exhibit strong antioxidant, antimicrobial and immunomodulatory features (Hooda et al. 2024; Yahfoufi et al. 2018). In this study, the nettle (*U. dioica*) extract was characterized mainly by rutin trihydrate (43,625 µg/kg) and catechin (10,997 µg/kg), together with caffeic acid, trans-ferulic acid, ellagic acid, quercetin, and cinnamic acid. Similar phenolic constituents have also been reported previously for U. dioica extracts (Meridja et al. 2025; Orčić et al. 2014; Uğur and Güzel 2023). Green olive tree (*Phillyrea latifolia*) extract analyzed in the present work contained mostly rutin trihydrate (3120 µg/kg). Other phenolic compounds detected were luteolin, cinnamic acid and quercetin. Gori et al. (2020) also found rutin and luteolin in the *P. latifolia* extract. Similar to our study, Dallali et al. (2025) reported that they detected luteolin and quercetin in the *P. latifolia* extract. Moreover, Gori et al. (2021) found hydroxycinnamic acid derivatives in the leaves of *P. latifolia*, whereas Aydin (2023) reported hydroxycinnamic acid derivatives in the *P. latifolia* extract, which explains the cinnamic acid we detected (Vogt 2010). Nonetheless, phenolic compounds in plant extracts are influenced by diverse factors, including but not limited to solvent type, plant part (aerial parts, roots, etc.), growth stage, geographical area, temperature, irradiation, drought, harvest time during the year, and even harvest time during the day (Aydin 2023; Dallali et al. 2025; Gori et al. 2020; Repajić et al. 2021). Therefore, compounds and their amounts in a given plant extract vary across studies. These compositional differences may partly explain why nettle induced a broader cytokine response, whereas green olive tree extract produced a more selective and moderate immunomodulatory pattern in the present study.

Plant extracts may promote growth (Awad and Awaad 2017). However, that is not always the case. Some phytochemicals in plants can also cause growth retardation (Taştan and Salem 2021). Therefore, it is important to assess each plant for each species as their effects may differ. Responses to plant extracts may vary among fish species because of differences in physiology, immune responsiveness, metabolism, and tissue-specific sensitivity, all of which can influence how phytogenic compounds are processed and how immune parameters respond. For example, in this study, it was determined that growth parameters in all groups of fish fed with nettle (N) extract did not change. On the contrary, Bilen et al. (2016) stated that N supplementation increased weight gain (WG) and specific growth rate (SGR), and decreased feed conversion rate (FCR) in rainbow trout. Likewise, Awad and Austin (2010) found that adding N to the diet activated digestive enzymes in rainbow trout, thus providing significant improvements in WG and specific growth rate SGR values. Similarly, Saeidi et al. (2017) showed N supplementation in rainbow trout increased WG and SGR. Moreover, 30 days of green olive tree (GOT) extract supplementation in this study resulted in improvement of FCR. However, other growth parameter values, although slightly increased, were not significantly affected. These results indicate that neither extract produced a clear growth-promoting effect under the present conditions. However, green olive tree extract improved FCR at day 30, particularly in the GOT100 and GOT200 groups, suggesting a transient improvement in feed utilization during the early feeding period. Diverse results may be obtained across studies because the phytochemical content of plants, even within the same species, can vary significantly. Plant extracts may influence the intestinal microbiota, thereby exerting varying effects (Dezfoulnejad and Taravat 2022). For instance, certain phenolic compounds may stimulate growth performance by inhibiting various pathogenic or nonpathogenic bacteria in the intestine (Van Hai 2015).

Hematological assessment in fish is performed to detect changes resulting from nutrition, water quality, or disease (Fazio 2019). It is also a substantial indicator for overall fish health (Yilmaz et al. 2016). Routine hematological evaluation of fish includes total erythrocyte count (RBC), hematocrit (HCT), hemoglobin concentration (HB), and erythrocyte indices (MCV, MCH, MCHC). In this study, hematological parameters in the experimental groups were not affected by N supplementation. In the GOT trial, HCT and MCV increased, and MCHC decreased after 60 days of supplementation with 200 mg/kg of GOT extract. Contrary to these results, Awad and Austin (2010) found that the addition of 1% N to the feed significantly increased HCT and HB levels in rainbow trout. Another study by Saeidi et al. (2017) reported that N supplementation increased HCT, HB, lymphocyte, neutrophil and RBC values ​​in rainbow trout. Similarly, Mehrabi et al. (2020) demonstrated that N supplementation to rainbow trout resulted in significantly increased RBC, HB, and HCT levels. Almabrok et al. (2018) stated that, as in our first trial, lime tree (*Tilia tomentosa*) extract supplementation in carp did not affect hematological parameters (RBC, HB, HCT, MCH and MCHC). As inferred from the literature summarized, different extracts may yield varying results in different fish. The reason for this is the rich compound diversity in plant extracts. Nonetheless, without comprehensive research on each compound and their synergistic/antagonistic effects, it is unlikely to reach a conclusion about a responsible compound for the observed effects. In the present study, the increase in HCT in the GOT200 group occurred together with higher MCV values, while RBC and Hb remained unchanged. This pattern may reflect a limited hematological adjustment rather than a clear increase in oxygen-carrying capacity.

The use of medicinal plants containing bioactive compounds with immunostimulatory effects as feed additives promotes specific and nonspecific immune responses in fish (Ghosh et al. 2019). In the present study, respiratory burst activity increased significantly in gilthead seabream supplemented with 200 mg/kg nettle extract. Lysozyme and myeloperoxidase activities also changed significantly in both trials, although the response pattern differed between the two extracts. Respiratory burst activity reflects the oxidative killing capacity of phagocytic cells, lysozyme contributes to humoral antibacterial defense through the degradation of bacterial cell walls, and myeloperoxidase is associated with the microbicidal activity of phagocytic cells. In this respect, the increase in these parameters suggests an enhancement of innate immune responsiveness, although the most functionally relevant dose differed between the two extracts. In the nettle trial, the N100 group showed the clearest increase in lysozyme and MPO, whereas in the GOT trial lysozyme increased at GOT100 and MPO at GOT200 and GOT400. In agreement with these results, Awad et al. (2015) reported that dihydroquercetin obtained from deodar (*Cedrus deodara*) enhanced RBA in gilthead seabream. Similarly, Barka (2019) found that supplementation with GOT extract increased LYS and MPO activities in rainbow trout. Moreover, Saeidi et al. (2017) reported that N extract supplementation caused elevated LYS and RBA in rainbow trout. Zare et al. (2023) also showed that N supplementation increased LYS levels in rainbow trout. Similar to our research, Bilen et al. (2019) found that hibiscus extract increased RBA, LYS, and MPO activities in seabream and seabass fed for 60 days.

IL-1β is a pro-inflammatory cytokine produced mainly by macrophages and other immune cells and plays an important role in innate immunity (Male et al. 2006; Sakai 1999; Sakai et al. 2021). In our research, both N and GOT supplementation significantly increased *IL-1β* gene expression in the spleen and intestine, at lower to moderate doses. Similar to these findings, Mehrabi et al. (2020) found that N-supplemented feeds led to increased *IL-1β* expression in rainbow trout, and decreased as the dose increased. In another research, Baba et al. (2018) showed that olive (*Olea europea*) leaf extract supplementation caused enhanced *IL-1β* expression in the spleen of rainbow trout. Furthermore, the study by Hoseinifar et al. (2018) revealed that *Eriobotrya japonica* leaf extract supplementation increased the *IL-1β* expression in common carp. On the other hand, it was reported that 75 days of feeding with vegetable (soybean and flaxseed) oil-supplemented feeds did not affect the *IL-1β* expression levels in seabream (Montero et al. 2010).

*IL-6* is a cytokine synthesized immediately in response to inflammation (Wen et al. 2020), making it a key part of the innate immunity. In our research, with resemblance to the *IL-1β* findings, *IL-6* gene expression levels significantly increased in both organs in both trials. However, in some groups in the first trial, it was observed that expression levels had sudden downfalls. For example, in the nettle trial, *IL-6* expression levels in the spleen elevated as the concentration increased, but decreased at the 400 mg/kg concentration. Likewise, in the intestine, after the sevenfold increase in the 100 mg/kg group, *IL-6* expression decreased in the 200 mg/kg group in comparison with the 100 mg/kg group. However, it remained significantly higher than the control group, suggesting that N supplementation stimulates *IL-6* gene expression levels in gilthead seabream. Moreover, GOT supplementation showed a similar trend, indicating that GOT supplementation also promotes *IL-6* expression. In line with our findings, Mehrabi et al. (2020) found that N supplementation significantly increased *IL-6* gene expression in rainbow trout.

*IL-10* is a critical anti-inflammatory cytokine produced after proinflammatory mediators (Wen et al. 2020). N supplementation increased *IL-10* gene expression in the spleen and intestine of gilthead seabream. In the GOT trial, *IL-10* expression levels in the spleen significantly increased in the 100, 200 and 400 mg/kg-supplemented groups, as well as in the intestine in the 400 mg/kg-supplemented group. When comparing the expression levels of *IL-1β*, *IL-6*, and *IL-10*, significant differences are observed between the two trials. Nettle-supplemented groups showed approximately 123-fold increase in *IL-1β*, 167-fold increase in *IL-6*, and 74-fold increase in *IL-10*. On the other hand, these values were roughly 4, 10.5, and 3.5 for the GOT-supplemented groups, respectively. Within the limits of these independently conducted trials, nettle was associated with a stronger splenic cytokine response profile than green olive tree extract.

In this study, *TNF-α* expression increased in both examined tissues of nettle-supplemented fish, particularly at 200 and 400 mg/kg. In contrast, the response to green olive tree extract was more limited, with reduced splenic expression in several groups and a more variable pattern in the intestine. Similar *TNF-α* responses after dietary plant supplementation have been reported in previous fish studies, although the direction and magnitude of the response appear to depend on dose, tissue, and extract composition (Mehrabi et al. 2020; Zemheri-Navruz et al. 2019; Baba et al. 2018).

Taken together, the cytokine results indicate that nettle and green olive tree extract generated distinct immune response patterns in gilthead seabream. Since the two experiments were conducted independently, differences between nettle and green olive tree extract are better interpreted in terms of overall response profile than as direct one-to-one differences in fold-change magnitude. In nettle-fed fish, the strong upregulation of *IL-1β*, *IL-6*, and *IL-18*, together with the increase in *TNF-α* at higher doses, points to a broader activation of inflammatory and innate immune pathways, especially in spleen tissue. However, the simultaneous increase in IL-10 suggests that this activation was accompanied by counter-regulatory signaling, indicating an induced but still modulated response rather than a purely uncontrolled inflammatory state. In contrast, green olive tree extract produced a more limited and selective cytokine response. Although *IL-1β* and *IL-6* were upregulated, the reduction in splenic *IL-18* and *TNF-α* suggests that GOT did not trigger the same breadth of inflammatory activation as nettle. This may indicate a more regulated immunomodulatory profile. In the nettle trial, the overall response also showed a nonlinear dose pattern, suggesting that increasing supplementation did not result in a proportional increase in functional benefit. Importantly, the groups showing the strongest overall cytokine activation were not the ones with the highest survival. The N100 group showed the best protection after challenge together with elevated lysozyme and MPO activities, whereas some higher-dose groups showed stronger responses for several cytokines without a comparable improvement in survival. This pattern suggests that protection may have depended less on the intensity of cytokine induction alone and more on how effectively that response was translated into functional innate defense. The tissue distribution of the response also appeared to differ between the two extracts. Nettle induced a much stronger splenic cytokine response, suggesting a more pronounced systemic immune activation, whereas the response to green olive tree extract remained more limited and selective.

Challenge tests are widely used to evaluate the practical effects of dietary additives on disease resistance. In the present work, after 60 days of feeding, nettle-supplemented groups were subjected to a challenge test by being infected with *Vibrio* (*Listonella*) *anguillarum*. Subsequently, it was observed that all supplemented groups showed significantly higher survival rates (SR), with the highest being the 100 mg/kg-supplemented group (96% SR). Likewise, Mehrabi et al. (2020) achieved an 86% SR in nettle-supplemented rainbow trout infected with *Saprolegnia parasitica* (the control group was reported to have a 56% SR). Similarly, Baba et al. (2018) observed enhanced SR in rainbow trout supplemented with olive leaf extract. These findings suggest that nettle supplementation at 50–400 mg/kg feed provides protection against *V*. *anguillarum* infection.

Although this study comprehensively assessed the immunostimulatory effects of nettle and green olive tree extracts in gilthead seabream, several limitations should be considered. Due to technical problems, respiratory burst activity could not be measured and a valid challenge result could not be obtained in the green olive tree extract group. Moreover, the two trials were performed at different times using different fish populations and separate control groups; therefore, they should be interpreted as independent experiments. Additional limitations relate to the molecular and feed-preparation procedures. Primer efficiencies were not experimentally validated, and formal reference-gene stability analyses were not conducted, indicating that the RT-qPCR data should be interpreted as supportive rather than conclusive molecular evidence. Furthermore, although the extract solutions were applied in a standardized spray volume and mixed stepwise before drying, actual post-coating inclusion levels were not analytically verified, and some degree of coating heterogeneity or leaching during feeding cannot be ruled out. While these issues do not undermine the overall interpretation of the study, they should be considered when assessing the precision of dietary extract delivery.

## Conclusion

In conclusion, dietary supplementation with nettle and green olive tree extract modulated several innate immune parameters and immune-related gene expression in gilthead seabream. Among the tested treatments, nettle at 100 mg/kg feed showed the most consistent functional benefit, as it was associated with increased lysozyme and myeloperoxidase activities and the highest post-challenge survival against *Vibrio* (*Listonella*) *anguillarum*. In contrast, green olive tree extract also produced clear immunomodulatory responses, including changes in selected cytokines and nonspecific immune parameters, but these findings were not supported by a valid challenge test and should therefore be interpreted with caution. Overall, the present results support 100 mg/kg nettle as the most promising inclusion level under the conditions tested, whereas the effects of green olive tree extract warrant further confirmation through phytochemical characterization and challenge-based validation.

## Data Availability

Data supporting the findings of this study will be made available upon reasonable request.
